# Quantifying the abundance and survival rates of island-associated spinner dolphins using a multi-state open robust design model

**DOI:** 10.1038/s41598-024-64220-3

**Published:** 2024-06-26

**Authors:** Liah McPherson, Janelle Badger, Kyleigh Fertitta, Madison Gordanier, Cameron Nemeth, Lars Bejder

**Affiliations:** 1 Marine Mammal Research Program, Hawaiʻi Institute of Marine Biology, University of Hawaiʻi at Mānoa, Honolulu, HI USA; 2 Cetacean Research Program, Pacific Islands Fisheries Science Center, NOAA Fisheries, Honolulu, HI USA; 3 Zoophysiology, Department of Bioscience, Aarhus University, 8000 Aarhus, Denmark

**Keywords:** Conservation biology, Population dynamics

## Abstract

Spinner dolphins (*Stenella longirostris *subsp.) occupy the nearshore waters of several Hawaiian Islands. Due to their constrained behavioral pattern and genetic isolation, they are vulnerable to anthropogenic threats. Their occurrence and behavior are well-described, yet a lack of data on their abundance and survival rates hinders optimal conservation action. Using design-based photo-identification surveys, this study estimated the abundance, apparent survival, and emigration of spinner dolphins off the Waiʻanae Coast of Oʻahu through multi-state open robust design (MSORD) and POPAN modelling. Eight seasonal field seasons, (two winter, spring, summer, and autumn) each comprised of six surveys of the study area, were completed during two consecutive years. Seasonal abundance estimates derived from the best fitting model ranged from 140 (± 36.8 SE, 95% CI 84–232) to 373 (± 60.0, 95% CI 273–509) individuals and were lowest during winter seasons. The MSORD estimated a survival rate of 0.95 (± 0.02 SE) and a Markovian pattern of temporary emigration. POPAN modelling estimated a super-population size of 633 (± 78 SE, 95% CI 492–798), reflecting the total number of individual dolphins that used the study area during the entire study period. Additional research on circum- and inter-island dolphin movements around and between Oʻahu and the Maui Nui region may shed light on both seasonal movement patterns and overall abundance for the Oʻahu/4-Islands stock. This work represents the first systematic mark-recapture effort to assess the abundance and survival rates of these highly exposed dolphins, providing valuable insights for conservation and management.

## Introduction

Information on the abundance, survival, and movement patterns of wildlife populations is important to understand their conservation status^1^. Repeated estimates of population abundance can provide insights into a population’s seasonal distribution^2,3^, trajectory^4,5^, and interactions with anthropogenic and environmental stressors^6,7^. Such knowledge is critical to informing data-driven conservation and management actions to protect vulnerable species^8,9^.

In the marine environment, apex predators such as cetaceans play crucial roles in maintaining ecological balance^10,11^ and serve as indicator species for monitoring ecosystem health^12^. Moreover, cetaceans hold considerable economic and cultural value^13–16^, yet face a wide range of threats from human activity. Cetaceans are sensitive to anthropogenic disturbance such as direct and incidental removal^17^, habitat degradation^18^ and human-induced behavioral change^6^. In remote island regions with high human density, anthropogenic impacts on cetaceans are of particular concern, as small island-associated populations often exhibit specialized behavior, low gene flow and high site fidelity^19–21^.

Island-associated spinner dolphins (*Stenella longirostris *subsp.) in Hawaiʻi exhibit rigid daily patterns of activity, socializing and resting in nearshore waters during the day, and moving offshore at night to feed on fish and squid from the mesopelagic boundary community^22,23^. Hawaiian spinner dolphins prefer daytime resting habitat with shallow, calm, sheltered water and sandy substrate where visual detection of predators is easier^21,22,24^. The protected western coastlines of the islands of Oʻahu (Waiʻanae) and Hawaiʻi Island (Kona) are particularly suited to spinner dolphins’ daily behavior pattern, as they are sheltered from the strong, predominate northeasterly trade winds and abut deeper-water foraging grounds where they prey on the mesopelagic scattering layer^23,25^.

Spinner dolphins throughout the Hawaiian Archipelago exhibit low gene flow^26^, and are partitioned into five distinct stocks: Midway Atoll/Kure, Pearl and Hermes Reef, Ni‘ihau/Kaua‘i, O‘ahu/4-Islands region and Hawaiʻi Island^27^. Strong social structure within groups coupled with geographic barriers (deep ocean channels) between islands, contribute to the genetic isolation of these stocks^26^. Their strict behavioral patterns and genetic isolation make Hawaiian spinner dolphins vulnerable to considerable exposure from anthropogenic activities^21,22,26,28^.

Around Oʻahu, the Hawaiian Island with the highest human population density, there is cause for concern from pollution^29,30^, fisheries interactions^31,32^, and pathogens, e.g., *Toxoplasma gondii*, a protozeal disease recently identified in two stranded spinner dolphins^33^. Tourism activities focusing on cetaceans (e.g., swim-with and boat-based cetacean watching) can also cause adverse impacts^34,35^. Spinner dolphins’ predictability in coastal Hawaiian waters subjects them to a lucrative tourism industry. For example, in 2013, the average lifetime revenue generated by a single spinner dolphin off Oʻahu’s Waiʻanae Coast was estimated in excess > $3,300,00^16^.

Spinner dolphins off the Kona Coast of Hawaiʻi Island have the highest reported exposure rates to human activity of all cetacean species^35^. Specifically, dolphins are exposed to anthropogenic activities within 100 m for > 82% of the daytime, with a median duration of 10 min between exposure events^35^. Recent observations suggest that spinner dolphins less frequently use a previously preferred resting bay off the Kona Coast where they experienced the highest exposure rates to human activity^35,36^. However, this change in habitat use may be linked to the construction of aquaculture facilities outside the bay or aggression from bottlenose dolphins that frequent these facilities^37^. In response to concerns pertaining to the long-term impacts of tourism activities on spinner dolphins in Hawaiʻi, NOAA implemented federal legislation in 2021, prohibiting humans and boats from approaching spinner dolphins within 50 yards (45 m) within two nautical miles from shore (NOAA, 86 FR 53818). Further legislation is currently being considered by NOAA to restrict human access to critical dolphin resting bays (NOAA 86 FR 53844).

To effectively monitor cetacean populations and inform policymakers, reliable population parameter estimates are critical. For marine mammals, photo-identification and capture-recapture methods are widely used to estimate population parameters^38^. Photo-identification is used to identify individuals and develop capture histories for distinctive animals in the study population, from which encounter probabilities can be calculated and used to obtain estimates of population size, and survival and emigration parameters^39,40^. Collecting capture-recapture data under Pollock’s Robust Design (PRD), where multiple sampling sessions occur in quick succession within a primary sampling period, can provide more accurate estimates of parameters of interest^41^. Robust design models^42,43^ are widely used to estimate dolphin population parameters such as seasonal abundance, apparent survival, and temporary emigration^2,3^. Classically, models analyzing such data assume population closure within primary sampling periods, but model developments such as the multi-state open robust design model (MSORD) relax this assumption. The MSORD combines features of the robust design sampling and multi-state modelling, accounting for both heterogeneity caused by temporary emigration and imperfect detection probability. The MSORD allows animals to enter and exit a study area within a primary sampling period, relaxing the constraint of geographic closure and allowing higher accuracy and precision in its estimates^44–46^. This model has been increasingly used to estimate population parameters for cetaceans and other marine species with high rates of transience and temporary emigration^47,48^.

As spinner dolphins off Waiʻanae can travel long distances in a day and are known to use other coastlines of Oʻahu^24,49,50^, the MSORD better suits these dolphins’ behavioral ecology. However, in MSORD modeling, abundance can only be estimated within, and not across, primary periods. To estimate the total number of individuals using a study area throughout the entire study period, or the super-population, models such as the covariate based open Jolly-Seber (hereafter POPAN) model must be used^38,51^.

While some information on the resting and daily movement patterns of spinner dolphins off the Waiʻanae Coast is available^24,52^, there is a data deficit on their abundance and survival rates despite growing concerns for the population’s trajectory. A previous study estimated abundances of 149 individuals (± 18 SE, 95% CI 117–189) for June and July 2002 and 330 individuals (± 16 SE, 95% CI 300–362) for July, August, and September 2007^49^. However, these results are more than a decade old, preventing the implementation of well-informed management decisions. Additionally, this prior study did not follow a systematic survey design and used closed-capture models for parameter estimation^49^. The present study was designed with specific regard to the behavior and biology of Waiʻanae Coast spinner dolphins and followed a systematic mark-recapture approach. The aim of this study was to assess the abundance, apparent survival, and transition probabilities for these highly exposed dolphins, and provide reliable information to aid in stock management and monitoring of the population’s trajectory.

## Results

### Survey effort and summary statistics

**Figure 1:**
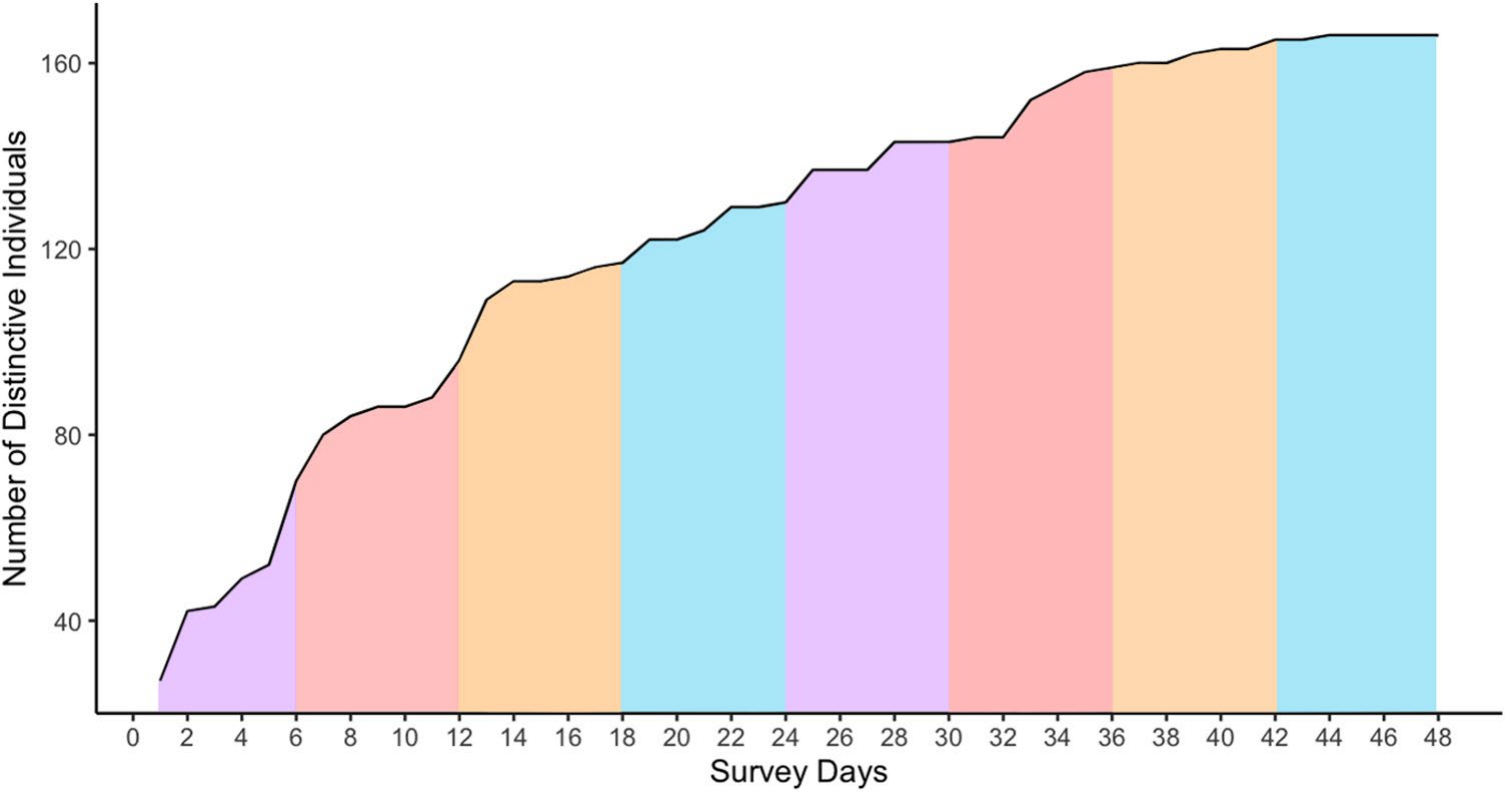
Cumulative discovery curve of marked individuals (distinctiveness D1 and D2). Field seasons are indicated by color (spring = purple, summer = red, autumn = orange, winter = blue).

**Figure 2:**
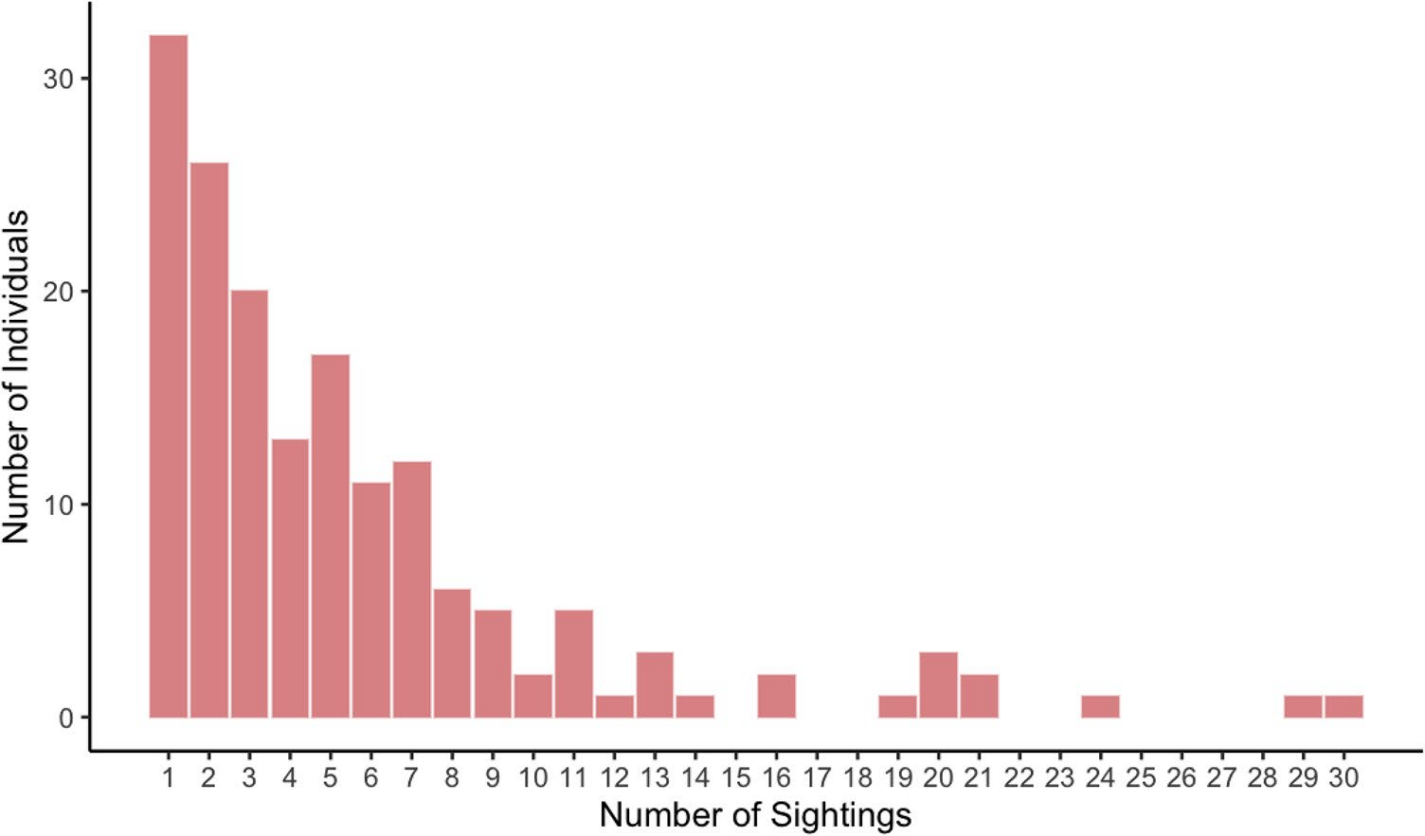
Sighting frequency of individual D1 and D2 spinner dolphins photographed between spring 2021 and winter 2023.

After an initial pilot season in winter of 2021, eight seasonal primary periods were completed between spring 2021 and winter 2023, each consisting of six secondary periods. Surveys for each secondary period were completed in one day, resulting in the inclusion of 48 secondary periods in the MSORD models. Survey days were chosen within a primary period dependent on weather and sea conditions to ensure good sightability. The mean number of days between consecutive secondary periods and consecutive primary periods were 2.9 (± 0.49 SE) and 78.3 (± 2.6 SE) respectively, and the average length of a primary period was 14.6 days (± 2.5 SE). Over the course of the study, 116 groups of spinner dolphins were encountered, with the highest numbers of sightings occurring during summer field seasons (Table 1). Dolphin groups were sighted along the entire Waiʻanae Coast, with concentrations around Kahe Point, Pokai Bay, and Makua/Yokohama Bay (Supplementary Figs S1). One hundred and sixty-five distinct dolphins (D1 and D2) were identified (Fig. 1). Sighting frequencies for individual dolphins ranged from 1 to 30, and 81% of dolphins were photographed on more than one occasion (Fig. 2). On average, dolphins were photographed five times (± 0.42 SE) throughout the study.

**Table 1:** Summary statistics for primary periods, including survey period, number of survey days, total number of groups sighted, and average group size.

Primary periods	Survey period	Survey days	No. of group sightings	Avg. group size
Spring 2021	9–19 Apr	6	19	40 (SD = 23.8)
Summer 2021	8–17 Jul	6	21	25 (SD = 16.7)
Autumn 2021	16–23 Oct	6	12	24 (SD = 13.0)
Winter 2022	5–20 Jan	6	7	36 (SD = 23.4)
Spring 2022	30 Mar–23 Apr	6	9	37 (SD = 29.4)
Summer 2022	6–22 July	6	21	35 (SD = 24.9)
Autumn 2022	10–20 Oct	6	18	21 (SD = 25.9)
Winter 2023	8 Jan–2 Feb	6	9	67 (SD = 32.3)

### Calculations of mark rates

Of the 116 dolphin groups sighted, 74 groups contained > 20 dolphins. D1 and D2 individuals in high quality images from these sightings were used to calculate Mark Rate 1 (θ_1_) (Supplementary Table S1). Forty-two groups contained ≤ 20 dolphins, and were used to calculate Mark Rate 2 (θ_2_). Calculations of the two mark rates produced similar estimates of the proportion of identifiable individuals in the population (θ_1_ = 0.30 ± 0.004 SE, θ_2_ = 0.30 ± 0.02 SE).

### Model selection

The most parsimonious MSORD model based on selection by AICc score had Markovian emigration, constant apparent survival and capture probabilities, and entry and persistence probabilities that varied by season and day (Table 2). No other models were within 2 AICc units of the best scoring model, though many parameter estimates were similar between top ranking models. A one-way ANOVA test showed no significance for the influence of swell ($p$ = 0.39) and sea state ($p$ = 0.56) on the number of dolphin groups sighted in each season, indicating that constant capture probability was plausible. Both the global-GOF test ($\sum \widehat{c}= 1.38$, ${\sum \chi }^{2}= 531$, $\sum df= 384$) and pooled secondary period tests (TEST 2: $\widehat{c} = 2.16$, ${\chi }^{2} = 26$, $df = 12$; TEST 3: $\widehat{c} = 2.09$, ${\chi }^{2} = 23$, $df = 11$) indicated good fit for the underlying data.

**Table 2:** The top six ranking models applied to the dataset ranked by AICc, change in AICc, AICc weight, and number of parameters.

Model	No. of parameters	AICc	∆AICc	AICc weight
${\varvec{S}}\left(.\right){\varvec{p}}\left(.\right){\varvec{p}}{\varvec{e}}{\varvec{n}}{\varvec{t}}({\varvec{s}}{\varvec{e}}{\varvec{a}}{\varvec{s}}{\varvec{o}}{\varvec{n}}.{\varvec{d}}{\varvec{a}}{\varvec{y}})\boldsymbol{\varphi }\left({\varvec{s}}{\varvec{e}}{\varvec{a}}{\varvec{s}}{\varvec{o}}{\varvec{n}}.{\varvec{d}}{\varvec{a}}{\varvec{y}}\right){\varvec{\psi}}({\varvec{M}}{\varvec{a}}{\varvec{r}}{\varvec{k}}{\varvec{o}}{\varvec{v}}{\varvec{i}}{\varvec{a}}{\varvec{n}})$	**69**	**3907.6**	**0.0**	**1.0**
$S\left(.\right) p\left(.\right) pent(season.day) \varphi \left(season\right) \psi (Markovian)$	47	3935.1	27.3	0.0
$S\left(.\right) p\left(.\right) pent(season.day) \varphi \left(.\right) \psi (Markovian)$	39	3964.7	57.1	0.0
$S\left(.\right) p\left(season\right) pent(.) \varphi \left(.\right) \psi (Markovian)$	23	4231.8	324.2	0.0
$S\left(.\right) p\left(.\right) pent(season) \varphi \left(t\right) \psi (Markovian)$	27	4234.3	326.7	0.0
$S\left(.\right) p\left(.\right) pent(.) \varphi \left(season\right) \psi (Markovian)$	23	4240.9	333.4	0.0

$S\left(.\right)$ = constant apparent survival; $p\left(.\right), p\left(season\right)$= capture probabilities that are constant or vary by season; $pent\left(.\right), pent\left(season\right), pent(season.day)$= entry probabilities that are constant, vary with season, or with season and day; $\varphi \left(.\right) , \varphi \left(t\right), \varphi \left(season.day\right)$= persistence probabilities that are constant, vary with season, or with season and day; $\psi \left(Markovian\right)$ = Markovian emigration. The most parsimonious model is signified by bolded text.

For POPAN modelling, the GOF test for the CJS global model ($\widehat{c}=2.13$, ${\chi }^{2} = 49$, $df = 23$) indicated overdispersion of the data. To reduce overdispersion, the models were adjusted by $\widehat{c}$ and ranked by QAICc score. The most parsimonious POPAN model had time varying capture probability, and constant apparent survival and entry probability. No other model was within two QAICc units of this top-ranking model.

### Seasonal and super-population abundance estimates

**Figure 3:**
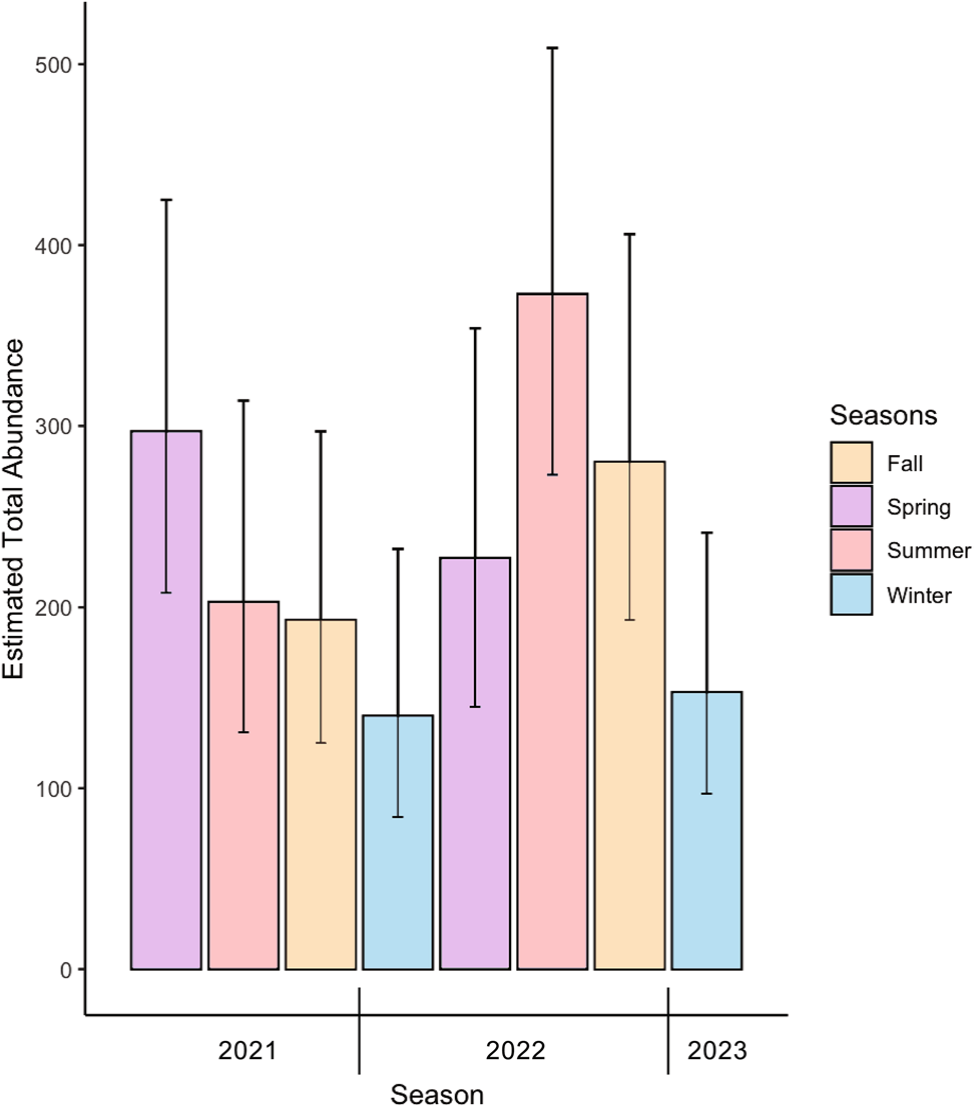
Estimated seasonal abundance (${\widehat{N}}_{total}$) and 95% confidence intervals for Waiʻanae Coast spinner dolphins derived from the most parsimonious MSORD model.

Seasonal abundance, an estimate of the total number of dolphins using the study area for each season, and super-population abundance, representing the total number of dolphins estimated to use the study area between April 2021 and January 2023, were calculated. Seasonal abundance estimates were calculated from the best fitting MSORD model (Table 3). Abundance was lowest in winter of 2022 (140 ± 36.8 SE, 95% CI 84–232) and highest in summer of 2022 (373 ± 60.0, 95% CI 273–509). Estimates of abundance were, on average, lowest during the two winter field seasons (Fig. 3). The total number of distinct individuals in the super-population estimated by the top ranking POPAN model was 190 (± 10.2 SE, 95% CI 170–210), resulting in a total super-population estimate of 633 (± 83 SE, 95% CI 491–817).

**Table 3:** Capture-recapture estimates of spinner dolphin abundance for all primary periods.

Primary period	n	${\widehat{N}}_{m}$ (SE, 95% CI)	${\widehat{N}}_{total}$ (SE, 95% CI)
Spring 2021	72	89 (3.5, 82–96)	297 (54.7, 208–425)
Summer 2021	48	61 (2.6, 56–66)	203 (45.6, 131–314)
Autumn 2021	49	58 (2.7, 53–64)	193 (43.1, 125–297)
Winter 2022	35	42 (2.0, 38–46)	140 (36.8, 84–232)
Spring 2022	46	68 (3.3, 62–74)	227 (52.3, 145–354)
Summer 2022	94	112 (3.1, 106–118)	373 (60.0, 273–509)
Autumn 2022	66	84 (3.1, 78–90)	280 (53.6, 193–406)
Winter 2023	43	46 (1.2, 44–48)	153 (35.9, 97–241)

***n*** = number of individuals identified, ${\widehat{N}}_{m}$ = estimated abundance of distinctive (marked) individuals, ${\widehat{N}}_{total}$ = estimated total abundance.

### Apparent survival and emigration

The rate of apparent survival between seasons from the best fitting MSORD model was 0.95 (± 0.02 SE, 95% CI 0.89–0.98). The probability of a dolphin being a temporary emigrant in primary period *t* if it was present in the study area in primary period *t − *1 ranged from 0.21 (± 0.10 SE) to 0.61 (± 0.8 SE), and the probability of being a temporary emigrant if absent in primary period *t *− 1 ranged from 0.27 (± 0.1 SE) to 0.91 (± 0.1 SE) (Supplementary Table S2). In all but one instance, the probability of a dolphin being a temporary emigrant from the study area given that it was previously absent (${\psi }^{E\to E}$) was higher than if it was previously present (${\psi }^{P\to E}$).

## Discussion

This is the first systematic mark-recapture study to assess the abundance and demographic parameters for spinner dolphins off Waiʻanae, Oʻahu. The sampling design was developed based on previous knowledge of the dolphins’ behavior and daily movement patterns to estimate abundance, apparent survival, and transition probabilities^22–24,49,50,52^. Data collected over two years resulted in the identification of 165 distinct dolphins, many of which were resighted throughout the duration of the study. The most parsimonious MSORD model revealed seasonal variation in abundance that was generally lowest in winter, a high rate of apparent survival, and Markovian emigration. Findings from this study provide up-to-date estimates to inform management and prompt further inquiry into the movement of spinner dolphins around Oʻahu and between neighboring island regions.

The two mark rate estimates (based on D1 and D2 animals) calculated by independent methods in this study are similar (θ_1_ = 0.30 ± 0.004 SE, θ_2_ = 0.30 ± 0.02 SE), and are within the range of other mark rates reported for coastal spinner dolphin populations in the Hawaiian Islands and elsewhere^49,53,54^. Mark rates for D1 animals ($\widehat{\theta }$_1_ = 0.07 ± 0.002 SE, $\widehat{\theta }$_2_ = 0.04 ± 0.01 SE) are lower than those reported for dolphins off the Kona Coast ($\widehat{\theta }$_1, Kona_ = 0.35 ± 0.02 SE, $\widehat{\theta }$_2, Kona_ = 0.36 ± 0.03 SE)^20^. However, an examination of a subset of D1 dorsal fins from the Tyne et al.^20^ catalog revealed a discrepancy in the cut-off between D1 and D2 animals compared to the cut-offs in the present study. Specifically, many fins in the Kona Coast population that were designated as D1s in the Tyne et al. (2014) study, would, by the NOAA PIFSC distinctiveness grading standards, be designated as D2s. Therefore, the mark rates in the present study are likely comparable to Tyne et al. (2014)’s mark rates for D1 animals.

The rate of apparent survival as estimated by the most parsimonious model was high and constant (0.95 ± 0.02 SE, 95% CI 0.89–0.98) between seasons. This estimate resembles that of spinner dolphins off Kona (0.97 ± 0.05 SE)^20^, however is it important to note that apparent survival in the present study was estimated seasonally instead of annually. High and constant survival is typical for dolphins and other long-lived species with slow reproductive rates^2,55^. This indicates little permanent emigration and low mortality, which are supported by individual capture histories throughout the study, yet the length of the study (two years) warrants cautious interpretation of long-term survival. Apparent survival is the product of true survival and permanent emigration and reflects true survival if permanent emigration approaches zero. Therefore, the apparent survival rate in this study may represent true survival if the results from two years of study are representative of longer time periods.

Seasonal abundance as estimated by the MSORD ranged between a low of 140 individuals (± 36.8 SE, 95% CI 84–232) in Winter 2021, to a high of 373 individuals (± 60.0 SE, 95% CI 273–509) in summer 2022. Estimates were generally higher mid-year and lower in winter. The only previously published abundance estimates for spinner dolphins off Waiʻanae fall within this range of abundances^49^, however, it is difficult to evaluate trends in abundance between the earlier efforts and results presented here given differences in assumptions and study designs.

The most parsimonious MSORD model revealed a Markovian pattern of emigration, meaning that an individual’s probability of being a temporary emigrant was dependent on whether the individual was an emigrant in the previous primary period. Generally, the probability of being a temporary emigrant during a primary period was higher if the individual was absent from the study area during the previous primary period. The highest transition probability of previous emigrants to the study area, derived as ($1-{\psi }^{P\to E}$), occurred between spring and summer 2022, reflecting the highest abundance estimate in summer 2022. Markovian emigration rates suggest that dolphins temporarily emigrate from and return to the study area during later, if not consecutive seasons in a structured movement pattern, however this structure was not determinable from only two years of data and may be further clarified with more years of sampling. The dynamic rates of temporary emigration (Supplementary Table S2) are not opposed with the indication of low permanent emigration suggested by the high apparent survival rate, as permanent emigration implies that dolphins never return to the study area. Information on the sex of dolphins in the population might additionally inform movement patterns, as males and females often exhibit differences in behavior and home range^7^.

Low seasonal abundance during winter 2021 (140 ± 36.8 SE, 95% CI 84–232) and 2022 (153 ± 35.9 SE, 95% CI 97–241) coincided with high rates of emigration from the study area prior to each winter season (Autumn → Winter 2021 ${\psi }^{P\to E}\hspace{0.17em}$= 0.67 ± 0.1 SE, 95% CI 0.5–0.8; Autumn → Winter 2022 ${\psi }^{P\to E}\hspace{0.17em}$= 0.54 ± 0.07 SE, 95% CI 0.40–0.67). The lack of variation in sighting conditions between seasons suggests that fewer dolphins use the Waiʻanae Coast during winter.

Seasonal patterns in abundance and movement are reported for many coastal dolphin populations including Commerson’s (*Cephalorhynchus commersonii*)^56^, Pacific white-sided (*Lagenorhynchus obliquidens*)^57^, pantropical spotted (*Stenella attenuata*)^58^, and bottlenose dolphins (*Tursiops *spp.)^3,59^. These patterns may be influenced by both intrinsic biological and environmental factors. For example, seasonal fluctuations in sea conditions and prey availability coincide with abundance in many delphinid populations, implying temporary emigration to exploit more suitable habitat and foraging conditions^56,60^. Off Bunbury, Australia, social dynamics and mating strategies in bottlenose dolphins drive sex-specific use of certain habitats and suggest that high summer and autumn abundance is linked to seasonal breeding aggregation^7^. In this study, differences between winter and mid-year dolphin abundance may be attributed to environmental variability and/or reproductive seasonality, though more data is needed for definitive conclusions.

The northeasterly trade winds in the Hawaiian Islands weaken between October and April, resulting in fluctuating wind patterns from other cardinal directions, increased rainfall, and bouts of southernly storms^61^. Additionally, during winter, extratropical storms in the North Pacific generate large-amplitude swells that propagate towards Hawai’i^62^. Swell, wind-waves, and runoff from rainfall can increase turbidity in coastal areas^63,64^. Consequently, the Waiʻanae Coast may experience increased turbidity and turbulent sea conditions during winter, while other coastlines around Oʻahu may be more sheltered. As spinner dolphins prefer clear, protected water for resting^65^, they may seek out these alternative coastlines more frequently during winter. This is consistent with results from aerial surveys from Hawaiʻi Island, which revealed a shift in spinner dolphin movement from the Kona Coast to the eastern side of the island during winter, particularly during episodes of southwesterly storms^22^. However, results from these aerial surveys must be interpreted cautiously, as the sample size was small and differences in sighting conditions on the leeward and windward coasts of this island may have influenced abundance estimates.

Seasonal variation in prey distribution might also drive trends in dolphin abundance. As spinner dolphins follow the diel horizontal and vertical migrations of their prey^23^, fluctuations in their abundance may mirror seasonal changes in the spatial distribution of the mesopelagic prey community. Future studies examining seasonal prey distribution and photo-identification of spinner dolphins from other coastlines of Oʻahu^50^ may further elucidate the reduced winter abundance of spinner dolphins off Waiʻanae.

Higher mid-year dolphin abundance may be partly explained by seasonality in their reproduction, with a potential influx of transient dolphins for breeding purposes. Patterns of reproduction in delphinids vary widely between species and regional populations, and can result in diffuse or concentrated calving periods^66,67^. While there are few data available on reproductive seasonality for Hawaiian spinner dolphins, inshore Eastern Tropical Pacific (ETP) (*Stenella orientalis*) spinner dolphins exhibit a calving peak between May and August^66^. Additionally, peaks in hormonal biomarkers such as testosterone and progesterone (indicating ovulation or pregnancy), in males and females, respectively, imply breeding seasonality^68,69^. Hormonal data from three captive Hawaiian spinner dolphins revealed elevated testosterone levels between March and September for one male dolphin, and a sharp increase in progesterone in late summer for two female dolphins^22^. The average gestation period for spinner dolphins is 10–11 months^22^, and evidence suggests higher numbers of neonate dolphins in summer and autumn off Waiʻanae (L. McPherson, personal observations), however neonates have been observed in all seasons. Therefore, it is possible that Hawaiian spinner dolphins have a diffuse peak breeding season mid-year.

Bottlenose dolphins in the vicinity of Bunbury, Australia, exhibit heightened seasonal abundance and an influx of dolphins during a pronounced peak breeding season^3,7^. Similarly, spinner dolphins off Waiʻanae demonstrate increased abundance and sighting frequencies during a potential mid-year breeding season. This period also witnesses a potential migration of transient dolphins to the study area. In other delphinid species, males possess larger home ranges or display higher dispersal rates compared to females, thereby affording them enhanced mating opportunities^70,71^. Among the 31 unique spinner dolphins observed only once in this study, the majority were sighted during the summer months (n = 13), with only one sighting recorded in winter. As spinner dolphins inhabiting the Oʻahu region are genetically similar with those found in Maui Nui^26^, it is plausible that some level of transience occurs between these two regions. Indeed, movement of spinner dolphins between Hawaiian Islands has been documented, though infrequently^49^. Transient male dolphins from Maui Nui or an offshore population may potentially visit the study area for breeding purposes during the reproductive season. However, the lack of available information regarding the sex of individual dolphins restricts further interpretation of these findings.

The POPAN estimate of super-population size (633 ± 83 SE, 95% CI 491–817) reflects the total estimated number of individual dolphins that used the Waiʻanae Coast between spring 2021 and winter 2023. A comparable estimate of the Hawaiʻi Island stock in 2011 (631, 95% CI 524–761)^20^ prompts further inquiry into variability in environmental and anthropogenic factors between the two island regions that might influence dolphin abundance and movement. Despite Tyne et al., (2014)’s suggestion that their 2011 estimate likely represented the entire Hawaiʻi Island stock, it is important to acknowledge that this stock stands out as the most genetically distinct within the Hawaiian Islands, indicating minimal emigration from the island. In contrast, dolphins off Waiʻanae belong to the Oʻahu/4-Islands stock and share genetic similarities with dolphins in the Maui Nui region. Therefore, the super-population estimate in the present study may represent abundance from a broader region than Oʻahu alone, yet additional information on transience and individual movement patterns could provide more insight.

The most recent abundance estimate of the Hawaiʻi Island spinner dolphin stock indicates a possible decline in abundance from previous estimates potentially due to decades of human disturbance^20,35^. Spinner dolphins around Oʻahu face various anthropogenic threats including chemical and noise pollution, harmful interactions with fisheries, and dolphin-centric tourism, yet there is a deficit of information on the population’s health and trajectory. Considering the possible declines off Hawai’i Island and the high vulnerability of Oʻahu’s spinner dolphins, it is important to establish long-term monitoring efforts to assess population health and inform conservation.

Investigation of the dolphins’ around-island movement patterns coupled with additional photo-identification data from other coastlines may prove valuable in determining if population estimates from Waiʻanae are representative of the island-wide population. Given the need for long-term monitoring, these explorations may indicate the viability of Waiʻanae as an indicator site for monitoring spinner dolphins around Oʻahu. Information on the potential transience of dolphins between Oʻahu and Maui Nui may also aid in the interpretation of the super-population size estimate and shed light on total abundance for the Oʻahu/4-Islands stock.

## Conclusions

This study provides systematic mark-recapture estimates of abundance and demographic parameters for the highly exposed spinner dolphins off Oʻahu’s Waiʻanae Coast and will aid in population monitoring efforts and stock management. The results indicate that (a) abundance estimates vary between seasons, with fewer dolphins using the study area during winter, (b) apparent survival is high and constant, indicating low permanent emigration from the study area, and (c) emigration is dynamic and Markovian. Low abundance of spinner dolphins off Waiʻanae during winter may be due to seasonal environmental conditions that reduce resting habitat quality. High mid-year abundance may indicate a breeding aggregation based on potential reproductive seasonality in Hawaiian spinner dolphins. Further research on the circum- and inter-island movement patterns of individual spinner dolphins may provide insight into total stock size, seasonal trends in abundance and best practices for long term monitoring.

## Methods

### Study area and sampling design

**Figure 4:**
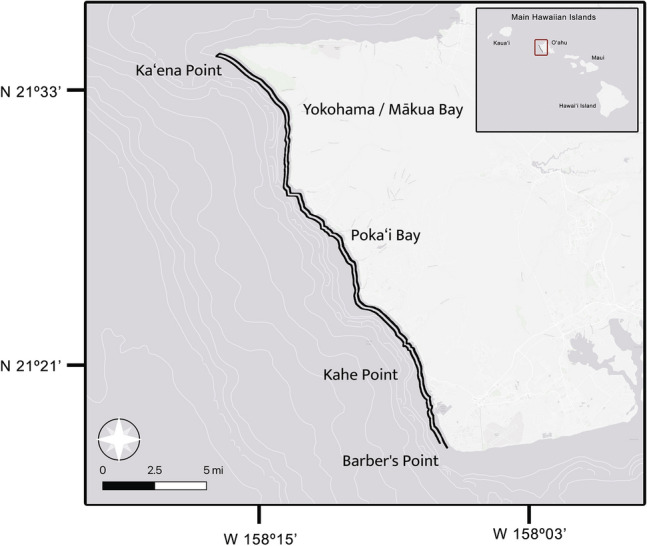
The study area covered approximately 40 km^2^ of coastline along the Waiʻanae Coast of Oʻahu, extending 1-km from shore between Barber’s Point and Kaʻena Point. Black parallel lines represent the survey transect route. This map and the inset of the Main Hawaiian Islands were generated in QGIS (QGIS Development Team, 2024. QGIS Geographic Information System. Open Source Geospatial Foundation Project. http://qgis.osgeo.org) and Adobe Photoshop (Adobe Inc., 2024. Adobe Photoshop. https://www.adobe.com/products/photoshop.html), respectively.

Boat-based surveys were conducted off the Waiʻanae Coast of Oʻahu, Hawaiʻi, departing from either Ko Olina or Waiʻanae marina covering ~ 40 km^2^ between Barber’s Point (21° 17.5′ N, 158° 06.5 W′) and Kaena Point (21° 34.5′ N, 158° 06.5′), extending 1-km from shore (Fig. 4). Water depth within the study area ranges from < 1 m near the coast to approximately 30 m at 1-km from shore. The deep-water edge off which spinner dolphins nocturnally feed is 2–3 km from shore, where the bathymetry quickly drops off to > 200 m. This area is the preferred habitat for spinner dolphins^65^, and their occurrence in this region is well-described^24^.

Boat-based photo-identification surveys were conducted during two consecutive years (starting in April 2021 and ending in January 2023) with seasonal efforts each winter (January), spring (April), summer (July) and autumn (October) onboard a 7 m research vessel equipped with twin 60hp outboard engines. Surveys were completed on the six best weather days within a season. The survey was designed to achieve complete coverage of the Waiʻanae Coast from the shoreline out to 1-km offshore (Fig. 4). Each survey team consisted of a boat captain and 2–4 visual observers. Acting under the assumption that observers sighted all dolphins within 250 m of the research vessel, two route configurations were trialed during a pilot season in Winter of 2021: a parallel-to-shore route and a zigzag transect route. The parallel configuration covered the entire coastline twice, with an inshore and offshore section, at 250 m and 750 m distance from shore, respectively (Fig. 4). The zigzag route was comprised of transect lines out to 1-km across the depth contour, randomly generated for each complete survey of the coastline. Trialing these two different survey configurations allowed a comparison of survey time and sighting effectiveness to optimize the design for future surveys. After the initial pilot season, the parallel-to-shore configuration was used for all remaining surveys.

### Photo-identification

Spinner dolphin groups were classified according to a 100-m chain rule, where any dolphins within 100 m of each other were considered to be in the same group^72^. During an encounter, two experienced members of the research team used Nikon D500 and Nikon D750 cameras equipped with either a 200 or 400 mm lens to photograph dolphins. Photographers targeted the dolphins’ dorsal fins for individual identification^73^, and were instructed to photograph animals randomly, to minimize bias towards distinctive fins. Observations of dolphin behavior and activity state were recorded for each encounter in addition to information about the group’s GPS location, current weather conditions, swell height, and sea state. Group size was estimated by observers on the vessel, and via unoccupied aerial system (UAS) when possible. Encounters ended when the team had high confidence for photographic coverage of the group or when weather/sea conditions deteriorated.

### Quality and distinctiveness grading of photo-identification images

Images of the spinner dolphins’ dorsal fins, which display unique marks and notches, were used to identify individuals^73^. Quantitative quality grading of dorsal fin photographs allowed a selection of images for analysis which minimized misidentification. Following protocols adapted from Rosel et al.^74^, photographs were graded by trained observers (n = 8) on a numeric scale for clarity, contrast, angle and visibility. Photographs were categorized as “excellent”, “good” or “poor”, the latter of which were excluded from analysis. Good and excellent quality photographs were then graded for feature distinctiveness, as not all fins were sufficiently marked to be included in the mark-recapture analysis. Distinctiveness was scored based on nicks and notches along the trailing and leading edges of the dorsal fin visible from both sides^74^. Highly distinctive fins with features evident in distant and poor-quality photographs were scored as D1, fins with two features or one major feature were scored as D2, fins with very subtle features were scored as D3, and completely clean fins were scored as D4. Photo grading was performed by multiple trained observers, and quality and distinctiveness scores were compared between observers for consistency.

### Estimation of the mark rate of the study population

Estimates of abundance in mark-recapture analyses relate only to the identifiable proportion of individuals in the population. To estimate total abundance, estimates are scaled to account for the nondistinctive proportion of the population. We estimated two mark rates following Tyne et al.^20^, by calculating mark rates ($\widehat{\theta }$_1_ and $\widehat{\theta }$_2_), using distinct (D1 and D2) animals.

Mark Rate 1 ($\widehat{\theta }$_1_, Eq. 1): The first mark rate was calculated with data from all groups of > 20 dolphins in which all animals in the group were photographed at sufficient quality for identification. Mark rate was estimated from the proportion of sufficient quality photographs that contained distinct fins.

1 $$\hat{\theta }_{1} = \frac{number\; of \;high \;quality \;images \;with \;distinct \;fins}{{total\; number \;of \;high \;quality\; images \;with \;distinct \;and\; non \;distinct\; fins}}$$

Mark Rate 2 ($\widehat{\theta }$_2_, Eq. 2): The second mark rate was calculated for groups with highly accurate group size estimates, and in which all dolphins in the group were photographed at sufficient quality for identification. This method was applied to photo-identification data from groups of ≤ 20 dolphins under the assumption that group size estimates would be accurate at or below this threshold.

2 $$\hat{\theta }_{2} = \frac{total\; number \;of\; distinct \;individuals \;in\; each \;group}{{sum of \;total\; group\; sizes}}$$

Standard error (SE) for each mark rate was calculated with Eq. (3):

3 $$SE\left(\widehat{\theta } \right)= \sqrt{\frac{\widehat{\theta } (1-\widehat{\theta } )}{n}}$$

where n is the sample size in each equation.

To estimate total abundance, an average of the mark rates calculated using method 1 and 2 was used in Eq. (4) to adjust the abundance estimate for the proportion of poorly marked and unmarked individuals:

4 $${\widehat{N}}_{total}= \frac{{\widehat{N}}_{m}}{\widehat{\theta }}$$

where ${\widehat{N}}_{total}$ is estimated total abundance, ${\widehat{N}}_{m}$ is the estimated abundance of marked individuals in the population, and $\widehat{\theta }$ is the estimated proportion of distinct individuals.

The standard errors for the total abundace estimate were calculated following the delta method^75^ in Eq. (5):

5 $$SE\left({\widehat{N}}_{total}\right)= \sqrt{{{\widehat{N}}_{total}}^{2}\left(\frac{SE{\left({\widehat{N}}_{m}\right)}^{2}}{{{\widehat{N}}_{m}}^{2}}+ \frac{1-\widehat{\theta }}{n\widehat{\theta }}\right)}$$

Log-normal 95% confidence intervals for the total population were calculated with the expression in Eq. (6):

6 $$C=exp\left(1.96\sqrt{ln\left(1+{\left(\frac{SE\left({\widehat{N}}_{total}\right)}{{\widehat{N}}_{total}}\right)}^{2}\right)}\right)$$

with a lower limit of $\frac{{\widehat{N}}_{total}}{C}$ and an upper limit of ${\widehat{N}}_{total} \times C$^76^.

### Multi-state open robust design modeling

The sampling approach of this study was specifically designed to follow Pollock’s Robust Design (PRD)^41^. An extension of the PRD capture-recapture model, the multi-state open robust design model (MSORD), was then used to estimate dolphin seasonal abundance, apparent survival, and temporary emigration^42,43,45^. The MSORD estimates temporary emigration as transition probabilities ($\psi$) between an observable ($P,$ present in the study area) and unobservable ($E,$ temporary emigrant; absent from the study area) state, modelling seasonal movement in and out of the study area (Supplementary Fig. S2). Primary sampling periods (here, seasons) are separated by long time intervals during which the population is assumed to be open. Within each primary period, multiple temporally close secondary sampling periods occur. In classical robust design modelling, the population is assumed closed within primary periods, whereas the MSORD model relaxes this constraint, allowing the population to be open^44,45^.

The MSORD assumes that (1) markings are permanent, unique, and accurately identified, (2) within a sampling occasion, capture probabilities are homogenous among individuals, (3) capture and recapture probabilities are homogenous, (4) the probability of capture for an individual is independent of other individuals, (5) sampling for secondary periods is instantaneous, (6) survival probabilities are equal between individuals, and (7) individuals may enter and exit the study area once during each primary period^41,45,77^. To minimize violation of assumptions one and two, only individuals with unique and permanent identifying features (D1 and D2) were used for analysis and only high quality (“good” and “excellent”) images were graded. Meeting assumptions three and four, capture and recapture probabilities were assumed independent and homogenous, as photo-ID is non-invasive and dolphins were not expected to exhibit behavioral response. Secondary sampling periods were completed in a single day to meet assumption five. Survival probabilities among age-classes may vary, however nearly all well-marked individuals appeared to be adults. Therefore, their survival probabilities between capture periods should be homogenous, minimizing violation of assumption six. It is possible that spinner dolphins entered or exited the study area more than once during a primary period, indicating potential violation of the seventh assumption. However, this is not expected to impact the results, as preliminary data from simultaneous surveys along adjacent coastlines^50^ do not show any such movement during secondary periods.

**Figure 5:**
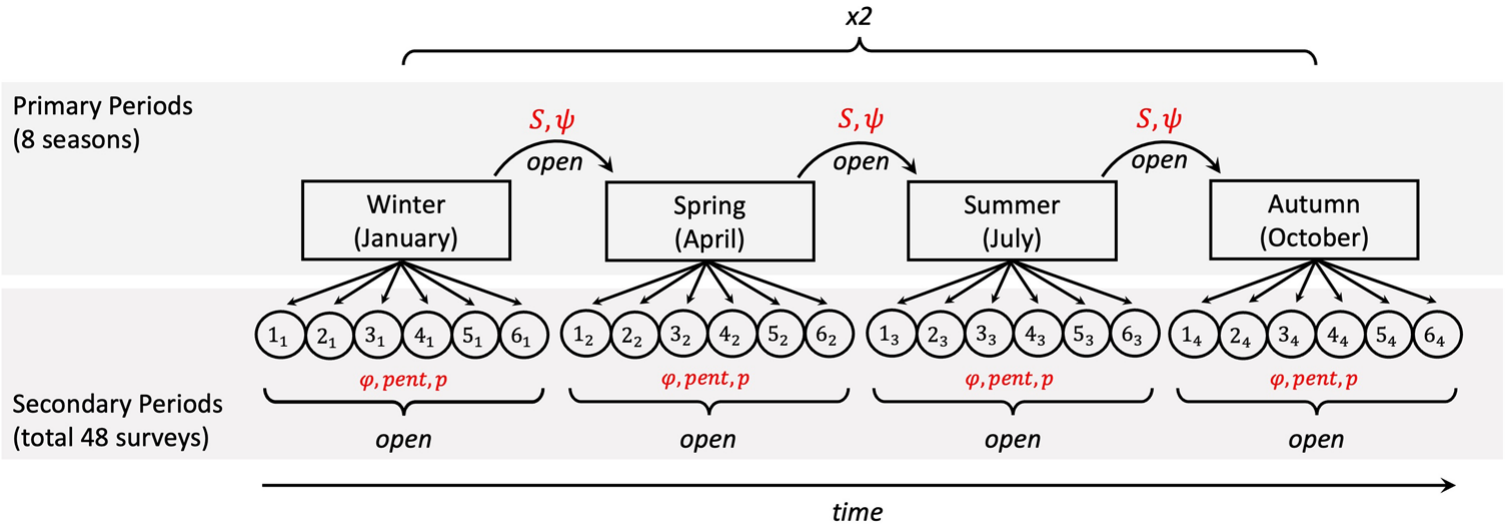
Survey sampling structure modelling the MSORD design, with population parameters in red. Primary periods occur seasonally over the course of two years, each of which contains six secondary sampling periods. Each secondary sampling period represents one complete survey of the Waiʻanae Coast.

The relationships between key parameters within the sampling structure of the MSORD model are shown in Fig. 5. Capture probability ($p$), the probability of an animal being detected given that it is within the study area, is estimated within primary periods. The probability of capture ($p$) and re-capture ($c$) are assumed equal ($p\hspace{0.17em}$= $c$), as photo-identification methods are non-invasive and should not elicit a “trap response”^1^. Entry probability ($pent$), the probability of entry into the study area during a given secondary period; and persistence probability ($\varphi$), the probability that an animal is within the study area during secondary period $j$, given that it was available in the area at period $j\hspace{0.17em}$− 1, are also estimated within each primary period. Between primary periods, apparent survival ($S$), which is the product of true survival and fidelity to the study area, i.e., the probability of surviving and remaining within the study area, and transition probabilities to the emigrant state (*E*) (${\psi }^{P\to E}$) and (${\psi }^{E\to E}$) are estimated. Transition probabilities between states sum to 1, therefore transition probabilities to the present state (*P*) are derived by subtraction (Supplementary Fig. S2). The MSORD also derives number of marked (here, distinctive) animals available in the study area (${N}_{m}$), and the average number of secondary periods that an animal stays within the study area during a primary period (residence time), however the latter was not interpreted for the purpose of this study. In the present study, one complete survey of the Waiʻanae Coast represents a secondary sampling period. Surveys were completed in one day to meet the assumption of instantaneous sampling as required by this design.

Robust design analyses were carried out using the program MARK^78^. Models were built in which the parameter estimate for apparent survival was held constant $S\left(.\right)$, or allowed to vary between primary periods $S\left(t\right)$. Transition probabilities were modeled with three patterns of emigration: random emigration, (${\psi }^{P\to E} = {\psi }^{E\to E}$), where the probability of an individual being an emigrant for a given primary period is *independent* of its absence during the previous primary period, Markovian emigration, (${\psi }^{P\to E}$ ≠ ${\psi }^{E\to E}$), where the probability of an individual being an emigrant is *dependent* on its absence during the previous sampling period, and no emigration (${\psi }^{P\to E} = {\psi }^{E\to E} = 0$). Capture probability was held constant $p(.)$, or allowed to vary with time between primary periods $p(season)$, or between both primary and secondary periods $p(season.survey)$. Entry and persistence probabilities were also held constant $pent(.)$, $\varphi \left(.\right)$, allowed to vary between primary periods $pent(season)$, $\varphi \left(season\right)$, or between both primary and secondary periods $pent(season.day)$, $\varphi (season.day$). Models with all combinations of these parameters were tested, and the Akaike Information Criterion with adjustment for small sample sizes (AICc) was used for model selection. All models within two AICc units of the model with the lowest AICc score were averaged if applicable^79^.

There are limited options for assessing parameter identifiability and goodness-of-fit (GOF) in MSORD models. First, data cloning was implemented to affirm that parameters were reliably estimable^80^. Here, the original dataset was cloned × 100 and fed to the suite of MSORD models, after which the standard errors of estimated parameters were examined. For this method, the parameter estimates should not change, but the standard errors for estimable parameters should converge at a rate of 1/$r$, $r$ being the number of times the dataset is cloned (in this case, 1/100)^80^. Models resulting in parameters with standard errors that did not converge at this rate indicated nonestimable parameters, and these models were excluded from the analysis. To test the underlying model assumptions, a time varying Cormack-Jolly-Seber (CJS) model^81–83^ was run on each primary period to calculate a global-GOF via average median $\widehat{c}$, where $\widehat{c}$ is defined as the deviance divided by the number of degrees of freedom. A second test examined GOF by pooling secondary periods such that dolphins were “seen” or “not seen” for each primary period and running a time varying CJS model in MARK’s program RELEASE^78^ to explore heterogeneity in capture probability (TEST 2 in RELEASE) and survival (TEST 3 in RELEASE).

### POPAN modeling

A suite of POPAN models were constructed to estimate the size of the super-population. POPAN models estimate parameters for apparent survival and entry probability in addition to super-population, however, only the super-population estimate was interpreted in this study, as parameter estimates for apparent survival and entry probability resulting from the MSORD model are likely more accurate due to its integration of movement dynamics^45,47^.

Captures of spinner dolphins were pooled within field seasons, so that each field season represented one sampling occasion. Capture probability ($p$), apparent survival ($S$), and entry probability ($pent$) were allowed to vary between sampling occasions ($season$) or were held constant ($.$). GOF was examined using program RELEASE in MARK, by calculating the average median $\widehat{c}$ for the fully time varying CJS model. As with the MSORD models, model selection was based on AICc score, or QAICc score when adjusted for overdispersion, and models within two AICc or QAICc units of the top-ranking model were explored^79^.

### Supplementary Information

Supplementary Information 1.

Supplementary Information 2.

Supplementary Information 3.

## Data Availability

The capture histories generated during this study are included in this article’s Supplementary Information files. Other data generated during and/or analyzed during the current study are available from the corresponding author on reasonable request.
