# Supplementary material for: Quantifying the abundance and survival rates of island-associated spinner dolphins using a multi-state open robust design model
Source: Sci Rep. 2024 Jun 26;14:14764. doi: 10.1038/s41598-024-64220-3 (PMC11208612; doi:10.1038/s41598-024-64220-3)
Supplement: Supplementary file 1 — Supplementary Information 1. [file 41598_2024_64220_MOESM1_ESM.docx]

Supplementary Material


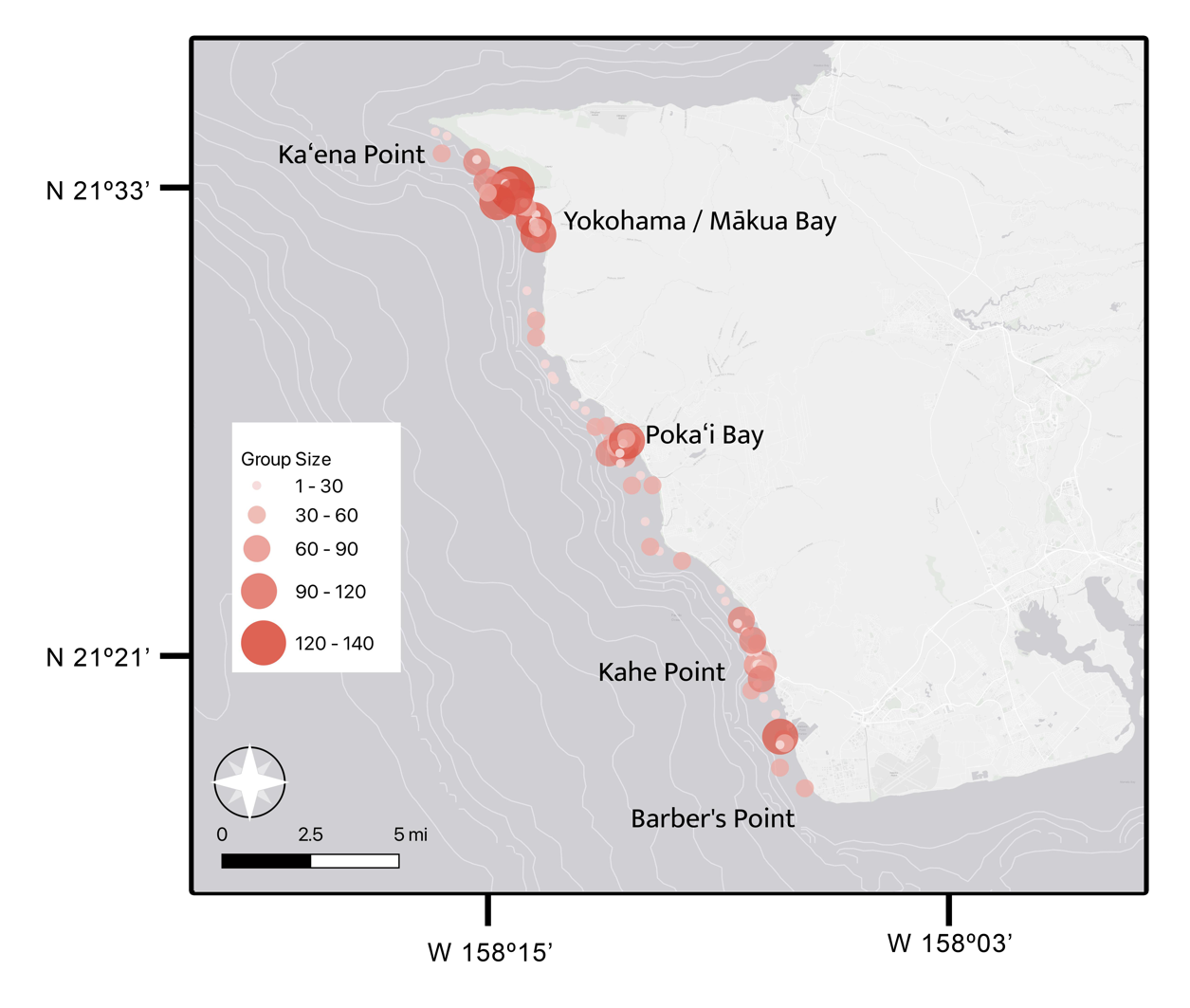


Supplementary Figure S1. Map of spinner dolphin sightings offshore Oʻahu’s Waiʻanae Coast during the eight-season study. This map was generated in QGIS (QGIS Development Team, 2024. QGIS Geographic Information System. Open Source Geospatial Foundation Project. <http://qgis.osgeo.org>).

Supplementary Table S1. Comparisons of mark rates calculated using two methods to measure the proportion of distinct dolphins in the population. Photo-identification data for calculations in this study came from three field seasons in 2021, four in 2022, and one in 2023.

|  | **Mark Rate 1 (θ_1_), Groups > 20 (SE)** | **Mark Rate 2 (θ_2_), Groups ≤ 20 (SE)** |
| --- | --- | --- |
| This Study: D1 | 0.07 (0.002) | 0.04 (0.01) |
| This Study: D1 and D2 | 0.30 (0.004) | 0.30 (0.02) |
| Kona Population: D1  (Tyne et al., 2014) | 0.35 (0.02) | 0.36 (0.03) |

Supplementary Table S2. Transition probabilities for dolphins emigrating from the study area ($\boldsymbol{\psi}^{\boldsymbol{P\to E}}$ **)** and remaining outside the study area ($\boldsymbol{\psi}^{\boldsymbol{E\to E}}$**)**. The highest and lowest transition probabilities are signified by bolded and italicized text respectively.

| **Primary Period** | $\boldsymbol{\psi}^{\boldsymbol{P\to E}}$ **(SE, 95% CI)** | $\boldsymbol{\psi}^{\boldsymbol{E\to E}}$ **(SE, 95% CI)** |
| --- | --- | --- |
| Spring 2021 $\to$ Summer 2021 | 0.61 (0.08, 0.46-0.74) | –– |
| Summer 2021$\to$ Autumn 2021 | 0.44 (0.10, 0.25-0.64) | **0.91** (0.08, 0.62-0.99) |
| Autumn 2021 $\to$ Winter 2022 | **0.67** (0.08, 0.49-0.81) | 0.81 (0.07, 0.65-0.91) |
| Winter 2022 $\to$ Spring 2022 | 0.38, (0.13, 0.17-0.65) | 0.65 (0.08, 0.48-0.80) |
| Spring 2022 $\to$ Summer 2022 | *0.21* (0.09, 0.08-0.44) | *0.27* (0.13, 0.09-0.58) |
| Summer 2022 $\to$ Autumn 2022 | 0.43 (0.07, 0.30-0.57) | 0.36 (0.29, 0.05-0.87) |
| Autumn 2022 $\to$ Winter 2023 | 0.54 (0.07, 0.40-0.67) | 0.83 (0.08, 0.61-0.94) |

Supplementary Table S3. Comparison of photo-identification efforts for spinner dolphins around Oʻahu since 2000.

| **Study** | **Study period** | **Survey days** | **Region** | **Mark Rate** | **Estimation method** | **Total abundance estimate** |
| --- | --- | --- | --- | --- | --- | --- |
| Present study | January 2021 – January 2023 | 48 | Waiʻanae Coast | 0.30 | POPAN | 627 (78 SE,  95% CI: 492-798) |
| Lacey et al., 2023 | ? | ? | All Oʻahu | NA | Distance sampling | 507 (95% CI: 260-987, CV = 0.35) |
| Hill et al., 2011 | July, August and September 2007 | 10 | Waiʻanae Coast | 0.46 | Closed capture | 149 (18 SE, 95% CI: 117-189) |
| Hill et al., 2002 | June – July 2002 | 11 | Waiʻanae Coast | 0.46 | Closed capture | 330 (16 SE, 95% CI: 300-362) |


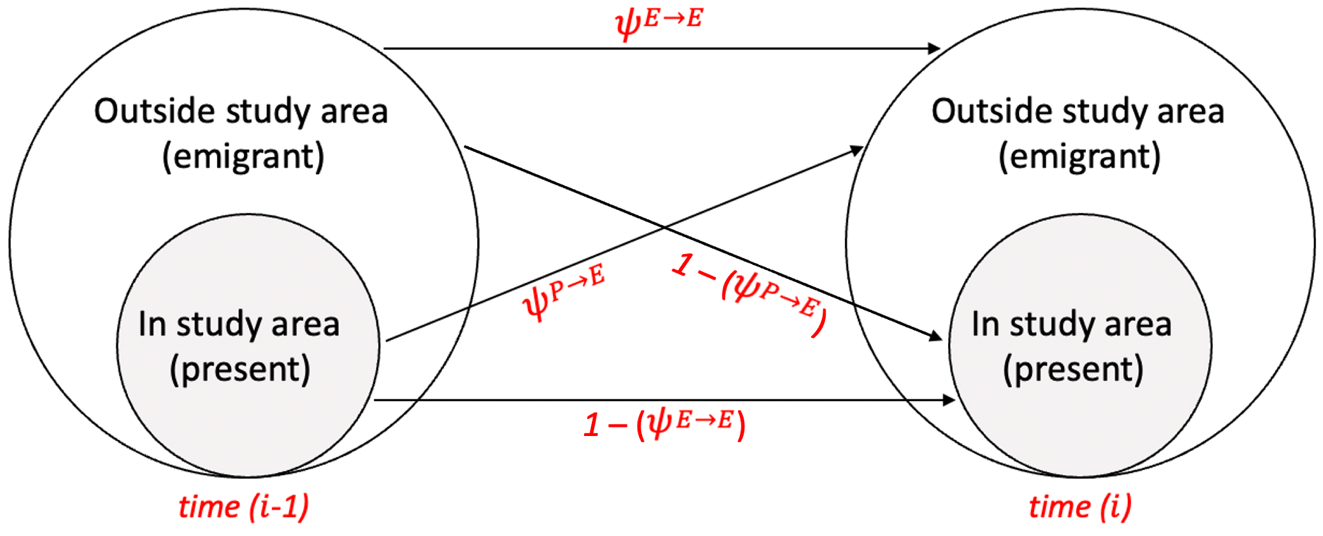


Supplementary Figure S2. Transition probabilities ($\psi$) between the observable ($P$) and unobservable ($E$) state for the MSORD model. Adapted from White and Burnham (1999)^76^.
